# Childhood malignancy and maternal diabetes or other auto-immune disease during pregnancy

**DOI:** 10.1038/sj.bjc.6600192

**Published:** 2002-04-08

**Authors:** L Westbom, A Åberg, B Källén

**Affiliations:** Department of Paediatrics, Lund University Hospital, SE-221 85 Lund, Sweden; Department of Obstetrics and Gynaecology, Lund University Hospital, SE-221 85 Lund, Sweden; Tornblad Institute, University of Lund, Biskopsgatan 7, SE-223 62 Lund, Sweden

**Keywords:** diabetes, childhood cancer, auto-immune disease

## Abstract

Among 4380 children born in 1987–1997 of women with a diagnosis of diabetes and alive at the age of one, 10 were registered in the Swedish Cancer Registry before the end of 1998. The odds ratio for having a childhood cancer after maternal diabetes, stratified for year of birth, maternal age, parity, multiple birth, and 500 g birth weight class was 2.25 (95%CI 1.22–4.15). Among 5842 children born during the period 1973–1997 whose mothers had other auto-immune diseases (SLE, rheumatoid arthritis, Crohn, ulcerous colitis, multiple sclerosis or thyroiditis), the number of observed childhood cancers (9) was close to that expected (8.5). Maternal diabetes but not other auto-immune diseases may be a risk factor for childhood cancer.

*British Journal of Cancer* (2002) **86**, 1078–1080. DOI: 10.1038/sj/bjc/6600192 www.bjcancer.com

© 2002 Cancer Research UK

## 

A few epidemiological studies have suggested that children whose families suffer from auto-immune disease including juvenile diabetes have an increased risk of haematopoietic malignancies (Till *et al*, 1979; Woods *et al*, 1987; Buckley *et al*, 1989). A Danish study (Mellemkjaer *et al*, 2000) revealed a moderately increased risk for childhood lymphomas and leukaemia of borderline statistical significance, while a positive association between childhood leukaemia and maternal diabetes was reported from Greece (Petridou *et al*, 1997).

Also, an increased risk of hospitalisation for childhood neoplasia was reported when the mother had diabetes before the pregnancy (odds ratio 1.64, 95%CI 1.06–2.54) but not when she had gestational diabetes (Åberg and Westbom, 2001).

Using the Swedish Cancer Registry we have examined the occurrence of cancer in children whose mothers had diabetes or other auto-immune diseases before the pregnancy, utilising the Swedish Registry of Cancer with a high diagnostic accuracy.

## MATERIALS AND METHODS

The study is based on a record linkage between two large and country-wide health registers in Sweden: the Medical Birth Registry and the Cancer Registry. The former contains medical information on practically all births in Sweden (1–2% are missing) and is based on copies of medical records from the antenatal care clinics, delivery units, and the paediatric examinations of the newborns (Cnattingius *et al*, 1990). Maternal diagnoses are given as ICD codes and women with diabetes can (since 1987) be identified from ICD code ICD9 648.0, ICD10 O24.0). Other auto-immune diseases could be studied from maternal diagnoses for the whole period 1973–1997: systemic lupus erythematosus (SLE), rheumatic arthritis (RA), scleroderma, Crohn's disease or ulcerative colitis (inflammatory bowel disease, IBD), multiple sclerosis (MS), and thyroiditis.

The Cancer Registry receives reports from physicians treating cancer patients and pathology and cytology departments, including autopsy findings. The overall reporting is estimated to be close to 99% (The National Board of Health, Stockholm, 1995) but lower registration rates may exist for specific types of cancer.

Linkage between the two registers was made using the unique identification numbers each person in Sweden receives and which is extensively used, including by all health care facilities.

In the study of maternal diabetes, births for the period 1987-1997 were followed in the Cancer Registry up to and including 1998. Four sets of data were identified: (i) all children born in these years with a diagnosis of childhood cancer; (ii) all children born to mothers with a diagnosis of diabetes and identified in the Cancer Registry; (iii) all children alive at the age of one born to mothers with a diagnosis of diabetes; and (iv) all children in the population alive at the age of one. The restriction to children alive at the age of one was made in order to compensate for the slightly higher infant death rate associated with maternal diabetes.

The risk for developing cancer in children whose mothers had diabetes was estimated as an odds ratio (OR), using Mantel–Haenszel procedure and stratifying for year of birth, maternal age (5 year classes), parity (1–4+), and sometimes also birth weight (500 g classes). Confidence intervals were determined using Miettinen's test based method (95%CI).

The effect of birth weight on cancer risk was also examined irrespective of maternal diabetes. A corresponding analysis for other maternal auto-immune diseases was made for the whole period 1973–1997.

## RESULTS

The data comprised details of 4380 children born of women with a diagnosis of diabetes and alive at the age of 1 year among 1 285 100 such children in the population, a prevalence of 3.4 per 1000.

We also identified 5842 children born to women with another auto-immune disease: 1505 with SLE or RA, 120 with scleroderma, 3945 with IBD, 102 with MS, and 122 with thyroiditis. A further 47 mothers had combinations of these diseases. The annual rate of identified cases varied markedly due to different ascertainment methods and use of different ICD classification systems. For the period 1973–1981, the mean annual number of cases was only 16, for the period 1982–1990 it was 135, and for the period 1991–1997 it was 639.

The total number of children, born in 1987–1997, registered in the Cancer Registry with a malignancy up to the end of 1998 and present also in the Medical Birth Registry, was 1419 (1.1 per 1000).

There were 10 children with cancer born of mothers with diabetes. Table 1

**Table 1:**
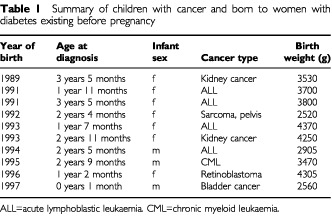
Summary of children with cancer and born to women with diabetes existing before pregnancy

lists these children with their cancers specified and their birth weight. Except for the last case, all children were at least 1-year-old at the cancer diagnosis. None of the 10 children was known to be diabetic. We checked the type of diabetes in the mothers of the 10 children with cancer: all had type 1 diabetes.

The expected number (irrespective of year of birth) of cancers among the children born to diabetic mothers is 4.8. Because of different follow-up times a stratification is required for year of birth resulting in an OR=2.19 (95%CI 1.19–4.02). If stratification is made also for maternal age, parity and multiple birth, the OR increases slightly to 2.38 (95%CI 2.15–2.62).

The impact of birth weight on cancer risk is shown in Table 2

**Table 2:**
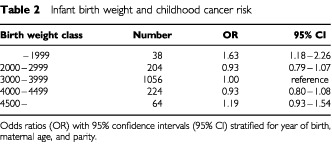
Infant birth weight and childhood cancer risk

. There is a statistically significant excess risk at very low birth weight (<2 kg) and a slight and statistically not significant increased risk at birth weight over 4.5 kg. The OR for childhood cancer given maternal diabetes, stratified for year of birth, maternal age, parity, multiple birth and 500 g birth weight class is 2.25 (95%CI 1.22–4.15). All 10 cancers were diagnosed before 4 years age. When the analysis is restricted to cancer diagnosed below age 4, the OR is 2.83 (95%CI 1.56–5.17) and when restricted to below age 2, the OR is 3.56 (95%CI 1.84–6.90).

Among the 10 infants with childhood cancer, seven were female, a sex ratio of 0.43 (exact 95%CI 0.07–1.87), thus not significantly different from the normal ratio of 1.06.

We identified nine instances of cancer among children born to women with other auto-immune diseases (Table 3

**Table 3:**
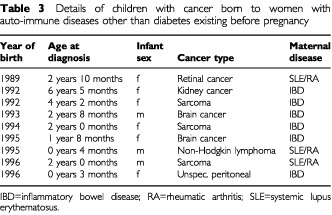
Details of children with cancer born to women with auto-immune diseases other than diabetes existing before pregnancy

) compared to 8.5, stratified for year of birth. It is of interest that there was no case of acute lymphatic leukaemia and that eight of the nine cancers were non-haematopoietic.

## DISCUSSION

The study was performed to explore an unexpected finding in a previous study, an increased hospitalisation rate due to neoplasms in children to mothers with diabetes mellitus before the relevant pregnancy (Åberg and Westbom, 2001).

The present paper is based on data from the national health registers in Sweden. It is to be expected that some cases of maternal diabetes are missed and also some cases of childhood cancer. The total rate of maternal diabetes observed (3.4 per 1000), however, agrees with the expected rate. Data loss must be non-differential as loss of information on maternal diabetes cannot be influenced by later childhood cancer and loss of cancer cases can hardly be affected by the presence of maternal diabetes. Such losses will therefore have very little effect on the risk estimates.

This study finds an increased risk of cancer among children whose mother had insulin dependent diabetes before the relevant pregnancy. This was apparently not an effect of macrosomia at birth. An increased risk of childhood cancer with high birth weight has been reported (Daling *et al*, 1984; Greenberg *et al*, 1985; Kaye *et al*, 1991; Petridou *et al*, 1997) and was also present in our material, though stratification for birth weight did not remove the statistically significant increase in OR. The observed increased risk at low birth weight was also found by Forsberg and Källén (1990).

We found no association between childhood cancer and maternal auto-immune diseases other than diabetes which weighs against a mechanism based on auto-immunity. A previously reported association between parental auto-immune disease and childhood cancer was mainly based on paternal diabetes (Mellemkjaer *et al*, 2000).

The risk increase seen with maternal diabetes amounts to about a doubling and is thus still very small. The contribution to childhood cancer in the population is also small due to the relatively low number of diabetic pregnancies. If one in 300 infants born have a mother with diabetes, this means that the total childhood cancer rate increases by 0.3%, about one case in each Swedish birth cohort (of approximately 100 000 births). It is possible that genetic factors are significant for the development both of cancer and of diabetes.
